# Methylcobalamin in Combination with Early Intervention of Low-Intensity Pulsed Ultrasound Potentiates Nerve Regeneration and Functional Recovery in a Rat Brachial Plexus Injury Model

**DOI:** 10.3390/ijms241813856

**Published:** 2023-09-08

**Authors:** Yueh-Ling Hsieh, Yu-Lin Lu, Nian-Pu Yang, Chen-Chia Yang

**Affiliations:** 1 Department of Physical Therapy, Graduate Institute of Rehabilitation Science, China Medical University, Taichung 40402, Taiwan; 2 Department of Physical Medicine and Rehabilitation, Hsin-Chu Branch, National Taiwan University Hospital, Hsinchu 30068, Taiwan; ul.family10@gmail.com; 3 School of Medicine, National Defense Medical Center, Taipei 11490, Taiwan; cmuhsieh@gmail.com; 4 Kao-An Physical Medicine and Rehabilitation Clinic, Taichung 40763, Taiwan; s901100@gmail.com

**Keywords:** brachial plexus injury, methylcobalamin, low-intensity pulsed ultrasound, functional recovery, spinal modulation

## Abstract

This study evaluated and compared the functional recovery and histopathological outcomes of treatment involving low-intensity pulsed ultrasound (LIPUS) and methylcobalamin (B12) on brachial plexus injury (BPI) in an experimental rat model. Three days after BPI, the rats were assigned to receive either LIPUS or methylcobalamin alone or in combination consecutively for 12 days. Serial changes in sensory and motor behavioral responses, as well as morphological and immunohistochemical changes for substance P (SP), ionized calcium-binding adapter molecule 1 (iba1), brain-derived neurotrophic factor (BDNF), and S100 were examined 28 days after BPI as the outcome measurements. Early intervention of LIPUS and methylcobalamin, whether alone or in combination, augmented the sensory and motor behavioral recovery as well as modulated SP and iba1 expression in spinal dorsal horns, BDNF, and S100 in the injured nerve. Moreover, the combined therapy with its synergistic effect gave the most beneficial effect in accelerating functional recovery. In view of the effective initiation of early recovery of sensory and motor functions, treatment with LIPUS and methylcobalamin in combination has a potential role in the clinical management of early-phase BPI.

## 1. Introduction

Brachial plexus injury (BPI) is one of the most disabling injuries of the upper extremity involving any degree of injury at any level of the plexus. Severe BPI occurs commonly in neonatal birth injuries, high-energy trauma, or motor vehicle accidents [1,2]. It is usually caused by sudden or prolonged traction or compression on the nerve roots that induces nerve ischemia, histopathological degeneration, and electrophysiological deterioration. Moreover, it often causes irreversible nerve dysfunction, resulting in prolonged functional impairment and flaccid palsy of the upper extremity [3]. Despite modern diagnostic procedures and advanced microsurgical techniques, functional recovery after brachial plexus repair is often unsatisfactory [4]. Therefore, strategies to accelerate axonal growth, maintain Schwann cells in a repair-capable state, and facilitate functional recovery are needed.

Methylcobalamin, the activated form of vitamin B12, is equivalent physiologically to vitamin B12 and may play important structural and functional roles in the nervous system. It was shown that nerve regeneration and functional recovery from peripheral nerve damage were significantly improved after methylcobalamin administration in experimental nerve injury models [5,6]. Methylcobalamin also promoted both in vitro differentiation of Schwann cells and in vivo myelination in a rat demyelination model [7], implying that methylcobalamin possesses a stimulating effect on proteosynthesis in Schwann cells at the initial stage of axon regeneration and it may facilitate neural regeneration [8]. Low-intensity pulsed ultrasound (LIPUS), which is one form of invasive physical stimulation, had therapeutic effects on promoting axonal regeneration in in vivo peripheral nerve injury trials [9,10]. It also increased the number of Schwann cells and promoted a greater number and larger area of regenerated axons in in vitro studies [11,12,13]. Moreover, LIPUS combined with nerve growth factor administration could also significantly increase the number of cells with neurites and average neurite length [14]. These findings indicate that methylcobalamin administration and LIPUS stimulation may have beneficial effects on peripheral neuropathy [10,15,16,17,18]. However, the synergistic mechanisms underlying the neuroprotective effects of LIPUS combined with vitamin B12 remain elusive.

Although initial-stage BPI is mainly treated conservatively, early rehabilitation still plays a fundamental role in the recovery after injury by shortening the time of neurodegeneration, improving its supporting matrix environmental conditions, and promoting axon regeneration whether receiving nerve reconstruction surgery or not [19]. The aim of the study was to evaluate the functional recovery and histopathological outcomes of independent treatment of methylcobalamin and LIPUS alone and in combination with BPI in an experimental rat model by examining serial changes in sensory and motor behavioral responses, as well as morphological and immunohistochemical changes in substance P (SP), ionized calcium-binding adapter molecule 1 (iba1), brain-derived neurotrophic factor (BDNF) and S100. This study hypothesized that early intervention of LIPUS mechanical stimulation combined with methylcobalamin micronutrient supplementation contributes to faster and greater functional recovery and nerve regeneration.

## 2. Results

### 2.1. Effects of LIPUS and Methylcobalamin on Motor Function and Sensory Behavioral Assessments in BPI Rats

#### 2.1.1. Sensory Behavioral Assessments

**Figure 1:**
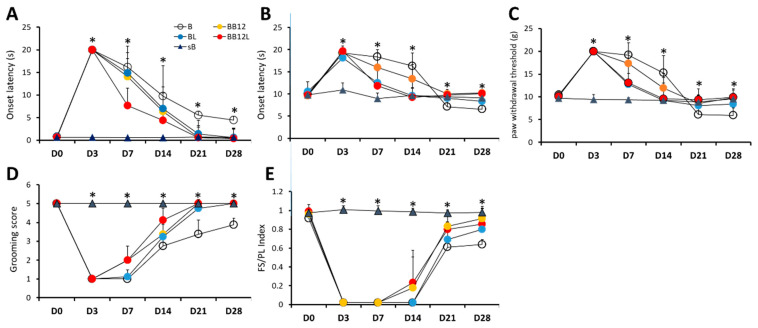
Effects of LIPUS and methylcobalamin alone or in combination on sensory and motor behavioral responses in B, BL, BB12, BB12L, and sB groups. Assessments of sensory thresholds were conducted by applying acetone (**A**), heat (**B**), and mechanical (von Frey filaments, (**C**)) stimuli to the palmar site of the front paw, corresponding to selective brachial territories. Assessment results of functional motor behaviors by grooming test (**D**) and walking track analysis (**E**) were expressed as scores and indices, respectively for the lesioned front limb. Recovery from BPI-induced hypoalgesia, hyposensitivity, and paralysis by either LIPUS or methylcobalamin alone or in combination was more prevalent. There were no significant differences among the four groups before surgery; * *p* < 0.05 indicates significant differences among the four groups by Kruskal–Wallis test.

Serial alterations of sensory thresholds on cold sensitivity tested using acetone stimulation, thermal sensitivity tested using radiant heat, and mechanical sensitivity tested using von Frey filaments at time points of D0, D3, D7, D14, D21, and D28 in five groups are shown in Figure 1A–C, respectively. As can be seen, the sB group (controls) showed insignificant changes in cold, thermal, and mechanical withdrawal thresholds throughout the experiment (*p* > 0.05, Table 1). In contrast, all BPI-operated groups, i.e., the B, BL, BB12, and BB12L groups showed significant time-dependent differences in these withdrawal thresholds at the six time points (all *p* < 0.01). After BPI (D3), a significant increase in onset thresholds of these paw withdrawal responses was observed in comparison with their values before BPI (D0) (D3 vs. D0, all *p* < 0.05). In acetone tests, after repeated application of LIPUS or methylcobalamin alone and in combination, the increases in cold withdrawal thresholds induced by BPI were reduced and returned almost to the baseline level by D21. No significant differences in cold withdrawal thresholds were found between D0 (before BPI) and D21 in the BL, BB12, and BB12L groups (all *p* > 0.05, Table 1), and between D0 and D28 in the BB12L group (*p* > 0.05, Table 1). BB12 and BB12L groups showed no significant differences in thermal and mechanical withdrawal thresholds at D14, D21, and D28 when compared with their values at D0 (all *p* > 0.05, Table 1). However, the B group still showed significant differences between each time point and D0; in particular, significantly shorter thermal and mechanical withdrawal thresholds at D21 and D28 than at D0 (all *p* < 0.05).

Prior to BPI (D0), none of the tests indicated significant differences in onset withdrawal latencies among the five groups (*p* > 0.05, Table 1). However, after BPI operation (D3), the cold, thermal, and mechanical withdrawal thresholds were markedly increased in the four BPI-operated groups compared with the sB group (*p* < 0.0001). All B groups had significantly prolonged cold withdrawal thresholds until the end of the experiment but significantly shorter thermal and mechanical withdrawal thresholds at D21 and D28 (*p* < 0.05). Only combined treatments significantly reduced the prolonged cold withdrawal thresholds in the BB12L group compared with the B, BL, and BB12 groups at D7 and D14 (all *p* < 0.01). Moreover, treatments with LIPUS or methylcobalamin alone and in combination significantly reduced the cold withdrawal thresholds prolonged by BPI in the BL, BB12, and BB12L groups starting from D21 (*p* < 0.001) to D28 (*p* < 0.001) compared with the B group. The present data also showed insignificant differences when compared with the sB group (*p* > 0.05). LIPUS and combined treatments significantly reduced the increased thermal and mechanical withdrawal thresholds at D7 and D14 when compared with either the B or BB12 group (all *p* < 0.05). Compared with the sB group, the BL, BB12, and BB12L groups showed no significant differences in cold, thermal, and mechanical withdrawal thresholds at D21 and D28 (all *p* > 0.05).

#### 2.1.2. Motor Function Assessments

Serial alterations of the scores and indices obtained from grooming test and footprints of the front limbs and forepaws on motor function for the five groups are shown in Figure 1D,E, respectively. As can be seen, the sB group showed no significant changes in grooming score and FS/PL index throughout the experiment (*p* > 0.05). In contrast, marked serial changes were observed in the B, BL, BB12, and BB12L groups (all *p* < 0.05). BPI significantly reduced the motor function of front limbs and forepaws compared with those treated with sham BPI at D3 (*p* < 0.0001). However, such loss of front limb grooming function induced by BPI improved after repeated application of LIPUS or methylcobalamin alone and in combination in the BL, BB12, and BB12L groups, with no significant differences in comparison with the B group at D21 (*p* > 0.05) and D28 (*p* > 0.05, Table 1). The grooming score was significantly improved starting from D7 (both *p* < 0.05) and the FS/PL index was significantly improved starting from D14 (both *p* < 0.05) in both the BB12 and BB12L groups when compared with the B group (Table 1). Moreover, the BL, BB12, and BB12L groups showed no significant differences in grooming scores at D21 and D28 when compared with that at D0 (all *p* > 0.05, Table 1), indicating a return to the baseline level. Moreover, only at D28 after combined treatment, the BB12L group showed no significant difference in FS/PL index in comparison with that at D0 (all *p* > 0.05, Table 1).

### 2.2. Effects of LIPUS and Methylcobalamin on Protein Levels of S100 and BDNF and Morphological Changes in Brachial Plexus of BPI Rats

**Figure 2:**
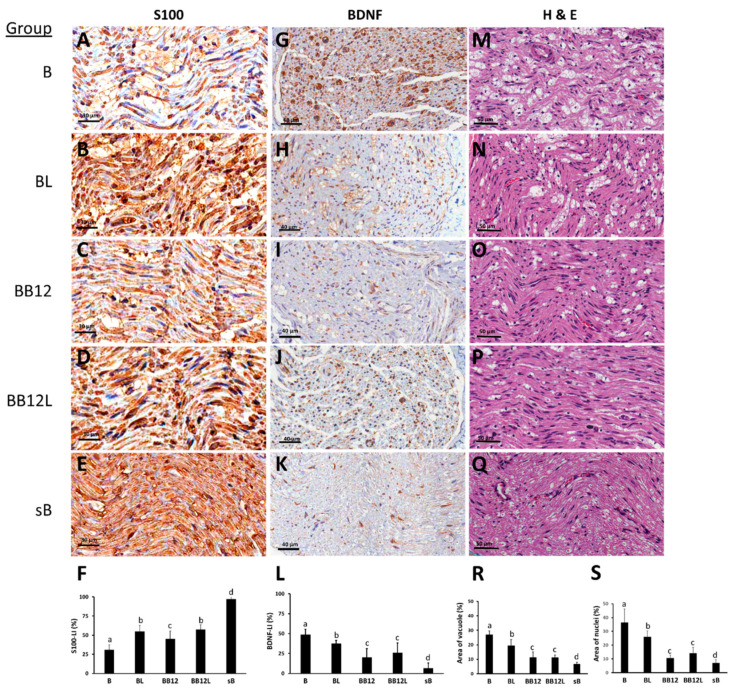
S100 (**A**–**E**), BDNF (**G**–**K**) immunohistochemical staining, and H&E (**M**–**Q**) staining in longitudinal cuts of nerves of representative animals. Data of area shown in percentage on S100-like immunohistochemistry (S100-LI, (**F**)), BDNF-like immunohistochemistry (BDNF-LI, (**L**)), vacuole formation (**R**), and inflammatory cell nuclei (**S**) are presented as mean ± SD. Values with different superscripts (e.g., a vs. b and b vs. c) indicate significant differences (*p* < 0.05) for all possible pairwise comparisons of means in injured sides of all groups tested by the Mann–Whitney U test.

Immunohistochemical analysis detected decreased S100-LI and increased BDNF–LI in all BPI-operated (B, BL, BB12, and BB12L) groups compared with the sB group (all *p* < 0.05). S100 protein was significantly weaker in the B group than in the BL, BB12, and BB12L groups (Figure 2A–F) and was clearly observed in the injured nerve (*p* < 0.05). S100 protein expression was higher in the LIPUS-treated groups (BL and BB12L groups) than in the group treated with B12 alone (*p* < 0.05).

BDNF-LI was mainly observed in the nuclei of the Schwann cells in all BPI-operated groups, and BDNF-LI expression was higher in the B group than in the sB group (*p* < 0.05). Moreover, the BL groups had moderate BDNF-LI while the BB12 and BB12L groups showed weak immunolabeling. In addition, immunohistochemical localization of BDNF was very weak in the sB group (Figure 2G–L).

The parameters of morphological changes such as inflamed cells and vacuole as evidenced by H&E staining in the five groups are presented in Figure 2M–Q, respectively. Morphological studies showed significant differences among the five groups (*p* < 0.05). After BPI, the B, BL, BB12, and BB12L groups had significantly more myelin-digestion chambers (vacuole formation) and increased inflammatory cell infiltration (inflammatory cell nuclei) compared with the sB group (all *p* < 0.05). An increase in both vacuole formation and nuclei of inflammatory cells was also significant in the B group compared with the BL, BB12, and BB12L groups (all *p* < 0.05). Moreover, infiltration of immune cells and BPI-induced vacuole formation were significantly decreased in the BB12 and BB12L groups compared with the BL group (*p* < 0.05), but the BB12 and BB12L groups showed no marked differences (*p* > 0.05, Figure 2Q,S).

### 2.3. Effects of LIPUS and Methylcobalamin on Protein Levels of SP and iba1 in Superficial Dorsal Horns of BPI Rats

**Figure 3:**
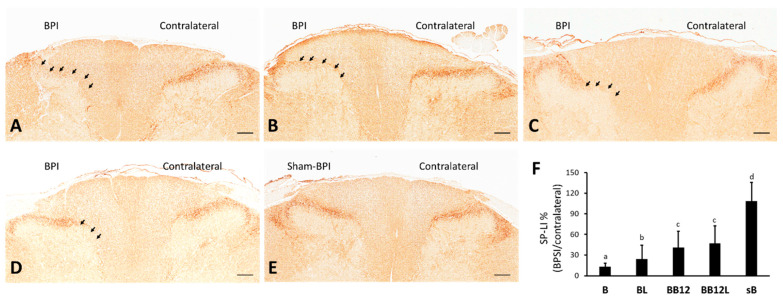
Substance P immunohistochemical (SP-LI) staining in dorsal horns of rats C5–C7. Partial depletion of SP confined to medial Laminae I and II (arrows) of B (**A**), BL (**B**), BB12 (**C**), and BB12L (**D**) groups. Normal SP is localized primarily across Laminae I and II in the sB group (**E**). Arrowheads mark areas with depletion of SP on the BPI side. Data of area shown in percentage on SP-LI (**F**) are presented as mean ± SD. Values with different superscripts (e.g., a vs. b and b vs. c) indicate significant differences (*p* < 0.05) for all possible pairwise comparisons of means in injured sides of all groups tested by the Mann–Whitney U test. Scale bars, 150 μm.

**Figure 4:**
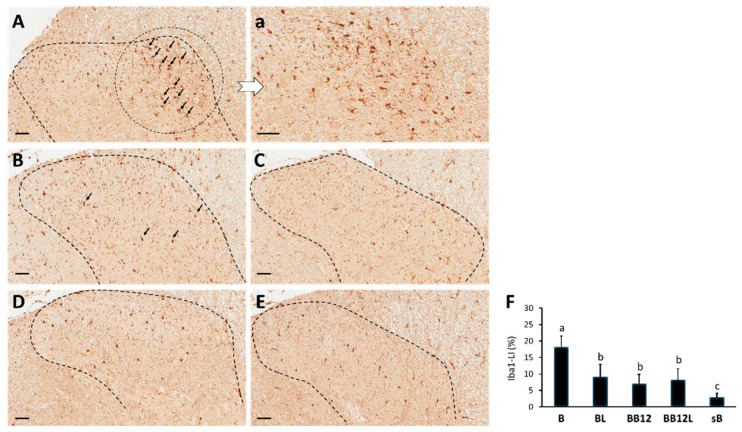
Representative iba1-like immunoreactivity (iba1-LI) in sections of dorsal horns of C5–T1 (dotted line). Distributions of iba1-LI staining area are mostly located in dorsal horns with significantly increased iba1-LI expression in dorsal horns observed in the B group (**A**,**a**) when compared with the BL (**B**), BB12 (**C**), BB12L (**D**) and sB (**E**) groups. Arrowheads mark areas with overexpression of iba1-LI on the BPI side. Data of iba1-LI (**F**) in dorsal horns are presented as mean ± SD. Values with different superscripts (e.g., a vs. b and b vs. c) indicate significant differences (*p* < 0.05) for all possible pairwise comparisons of means tested by Mann–Whitney tests. Scale bars, 50 μm.

Significant differences in the expression of SP-LI (Figure 3A–E) and iba1-LI (Figure 4A–E) in the spinal dorsal horn were observed among the five groups (both *p* < 0.001, Figure 3F and Figure 4F). Decreased SP-LI and increased iba1-LI were significant in all BPI-operated groups compared with the sB group (*p* < 0.001). SP-LI was more clearly observed in the BL, BB12, and BB12L groups than in the B group (*p* < 0.05). In addition, SP-LI protein expression was higher in the BB12 and BB12L groups than in the BL group (*p* < 0.05). There was significantly decreased iba1-LI expression in dorsal horns, especially in Laminae I and II, in the BL, BB12, and BB12L groups than in the B group (*p* < 0.001). However, there were no significant differences in the expression of iba1-LI among the BL, BB12, and BB12L groups (*p* > 0.05).

## 3. Discussion

This study utilized a rat brachial plexus defect model to demonstrate that the administration of LIPUS and methylcobalamin alone or in combination at the injury site improves sensory and motor functions after BPI. The three forms of treatment showed the following unique therapeutic effects for nerve regeneration and functional recovery. (1) LIPUS-included treatment (either LIPUS alone or LIPUS combined with methylcobalamin) at injured brachial plexus stumps initiates the recovery of thermal and mechanical sensation. (2) Methylcobalamin-included treatment (either methylcobalamin alone or methylcobalamin combined with LIPUS) at injured sites initiates the recovery of motor recovery of proximal (grooming) and distal (finger spreading) front limbs. (3) Combined treatment involving both LIPUS and methylcobalamin initiates the recovery of cold sensation. Taken together, these findings support the critical role of early intervention of LIPUS and methylcobalamin either alone or in combination in initiating functional recovery after BPI. Further morphological and immunohistochemical measurements at injured nerves also showed improvements, including inflammatory cell infiltration, vacuole formation, and BDNF expression reduced by methylcobalamin-included treatment, as well as S100 expression enhanced by LIPUS-included treatment. This novel approach of treating injured sites peripherally with LIPUS and methylcobalamin either alone or in combination could modulate spinal glial activation; however, only methylcobalamin-included treatment increased SP expression in dorsal horns.

Peripheral nerves regenerate at a rate of 1–3 mm daily and often must regrow over large distances, with axon extension dependent on the synthesis and transport of materials from the cell body [20]. Peripheral nerve injury involves complex molecular, cellular, and genetic events that help in ultimate nerve regeneration. Therefore, regeneration and reinnervation of the damaged peripheral nerve might also be obstructed by the downregulation of the natural regenerative mechanisms and chronic denervation that occurs over time. Delayed nerve regeneration will prolong neuron re-connection from the target and re-innervation of Schwann cells in the distal nerve pathways, which may cause distal nerve and end-organ atrophy over time and contribute to chronic sensory and motor dysfunction [21]. Although early nerve repair can result in improved functional outcomes, there are still no effective therapeutic methods for speeding up the regeneration rate due to the characteristics of axonal regeneration [22]. Therefore, even after optimal repair with advanced microsurgical procedures in the damaged peripheral nerve, there remains a need for diversified therapeutic adjuncts to accelerate the rate of peripheral nerve regeneration and functional recovery. This study used LIPUS and methylcobalamin both alone and in combination to promote nerve regeneration and recovery in a BPI rat model. It was found that although treatment with either LIPUS or methylcobalamin alone was already effective for nerve recovery, the combined treatment with both had an additive and synergistic effect on accelerating nerve regeneration and functional recovery.

Owing to slow axonal regeneration, structural changes in target organs, and an increasingly less supportive stromal environment for regeneration, the time frame for reinnervation of sensory receptors is much longer than that for motor nerves, often leading to incomplete sensory recovery [22]. Nevertheless, earlier repair still results in better sensory outcomes [23]. Sensory receptors can be reinnervated years after injury, but the maximum time frame remains uncertain. Moreover, various forms of physical stimulation are beneficial for promoting the recovery of sensory function, such as electric stimulation [24], magnetic stimulation [25], ultrasonic stimulation [26], and laser therapy [27]. Among these forms of physical stimulation, the thermal effect of LIPUS is minimal or absent due to very low intensities (≤100 mW/cm^2^) and the pulsatile nature of ultrasound. LIPUS, which causes minimal inflammation and no tissue damage, can thus be used in the acute phase of nerve injury for modulation of nerve regeneration and facilitation of drug delivery [18]. Compelling evidence demonstrated that the effect of LIPUS is mediated via the repair of Schwann cells to increase the number, diameter, or the myelination of axon distal to the lesion site, improve functional outcomes, and globally enhance peripheral nerve regeneration after nerve injury [18]. Previous studies have reported that daily exposure of the transected inferior alveolar nerve to LIPUS significantly promoted recovery of the head-withdrawal threshold in response to mechanical stimulation of the facial skin above the mental foramen when compared with LIPUS-unexposed rats [28,29]. These results suggested that LIPUS treatment accelerates the recovery of orofacial mechanosensory function following peripheral nerve transection [29]. However, studies investigating the effect of LIPUS on neuropathic pain and sensory recovery are still limited, with most of them focusing on motor outcomes. In this research, the administration of LIPUS and B12 either alone or combined significantly improved cold, thermal, and mechanical withdrawal thresholds in BPI rats. It was worth noting that rats under daily LIPUS treatment (either LIPUS alone or LIPUS combined with B12) at brachial plexus stumps showed earlier progress in increased cold, thermal, and mechanical sensitivities after BPI compared with LIPUS-unexposed rats. The present findings are consistent with previous results of animal studies [28,29] in that treatment with LIPUS alone or combined with B12 was effective in initiating recovery of thermo- and mechanosensory function after BPI; however, only combined treatment initiated cold sensation function. Moreover, rats receiving LIPUS alone had no sustained effect on the modulation of sensory function after cessation of LIPUS stimulation, but the effect could still be maintained in rats receiving the combined treatment even after cessation. It was speculated that the delivery and absorption of methylcobalamin may be facilitated by LIPUS, leading to the long-term effect.

It has been suggested that certain B vitamins support nerve regeneration and are thus called “neurotropic” vitamins because of their important functions in the nervous system, especially in maintaining the myelin sheaths [30]. Experiments on rats revealed that vitamin B12 promoted myelin formation and reduced Wallerian degeneration responses [31,32], showing a neuroprotective effect. Several studies demonstrated that vitamin B12 not only promoted significant functional recovery of the sciatic nerve but also thickened the myelin sheath in myelinated nerve fibers, and increased the cross-sectional area of target muscle cells [33,34]. A case study found that both motor and sensory functions of the upper extremity with BPI improved over 6 months after ultrasound-guided vitamin B12 injection into the injured nerve [35]. In addition, several studies revealed that high doses (0.5 mg/kg) of methylcobalamin delivered systemically or locally showed more significant improvement in nerve regeneration and functional recovery after nerve injury than low doses [6,7]. Similarly, in this study, treatment with methylcobalamin at high doses led to significantly faster recovery of motor skills in grooming and finger spreading, as well as decreased inflammatory cell infiltration and vacuole formation and increased expression of S100 protein in injured nerve fibers. Taken together, the present findings evidenced the neuroprotective effect of vitamin B12 after peripheral nerve injury.

Peripheral nerve lesions (axotomy) cause the death of a large number of dorsal root ganglion (DRG) cells, resulting in decreased synthesis of some neuropeptides, such as SP. DRG neurons decrease their SP synthesis; and SP levels in the dorsal horn also decline but return to normal if regeneration is successful. In adults, when regeneration is prevented, recovery of SP in the dorsal horn is slow and incomplete. Therefore, increased SP peptide synthesis by surviving axotomized DRG and dorsal horn accounts for nerve regeneration or sprouting from adjacent intact DRGs for recovery [36]. A marked quantitative decrease in SP in the spinal cord measured by radioimmunoassay was observed at 15 days after the rat sciatic nerve section [37]. In this study, increased SP expression in dorsal horns was observed after treatment indicating that the three treatment modalities, in particular, methylcobalamin and combined, may promote nerve regeneration. In addition, S100 immunoreactivity is predominantly found in myelin-forming Schwann cells, and the amount of S100 immunoreactivity in a Schwann cell correlates with the thickness of its myelin sheath formed [38]. The present finding of increased S100 protein expressed by Schwann cells after either LIPUS or combined treatment indicates an increased presence of myelinated axons for nerve regeneration or sprouting.

It has been suggested that increased BDNF synthesis in the lesioned peripheral nerve [39] and rapid activation and accumulation of microglia within the dorsal horn of the spinal cord where the injured sensory afferents terminate [40] might be associated with the initiation and persistence of neuropathic pain [41]. Previous results indicated that peripheral actions of BDNF might contribute to pain centralization and might also be involved in microglia–neuron interactions in the dorsal horn, highlighting its role in the periphery as a mediator of injury-induced neuronal hyperactivity and central sensitization [41]. Iba1, a microglia/macrophage-specific calcium-binding protein, has actin-bundling activity and participates in membrane ruffling and phagocytosis in activated microglia [42]. Prior research found that BPI induced overexpression of iba1 in dorsal horns of spinal cords corresponding to levels of the injured nerve on day 18 post-injury [43]. The present results revealed that LIPUS and methylcobalamin applied either alone or in combination significantly ameliorated the BPI-induced BDNF accumulation at the injured nerve and suppressed the overactivation of microglia via decreasing iba1 expression in dorsal horns. In addition, this study observed hyperalgesic responses in non-treated BPI-operated animals from D21 after injury. Methylcobalamin administered alone or in combination with LIPUS seemed to cause the most beneficial effects in suppressing the thermal and mechanical hyperalgesic responses, which may be correlated with decreased BDNF expression in injured nerves. These biochemical results suggested that the three treatment approaches studied reduced the development of neuropathic pain after BPI by modulating pain transmission and microgliosis, despite discrepancies between behavioral manifestation of pain and biochemical induction. In other words, the analgesia seen by biochemical expression, which is indicative of neuroplasticity changes in the pain pathway, may not be mirrored by the sensory behavioral assessments. These discrepancies, also reported in several studies, revealed that injured rats were under stress and might present behaviors inconsistent with the biochemical results [44,45,46].

LIPUS and vitamin B12 are administered for clinical treatment of nerve injury. LIPUS is a non-invasive physical modality frequently applied in the treatment of nervous system injury due to its mechanical stimulation effects, while vitamin B12 coenzyme is involved in a variety of main metabolic reactions. In this study, in addition to improvements in functional recovery and histopathological outcomes after independent treatment involving methylcobalamin and LIPUS alone, the combined treatment involving both seems to initiate earlier progress in these outcomes. The mechanisms underlying the combined use of LIPUS and methylcobalamin for treating BPI include initiating early regeneration, addressing the excessive activation of microglia, modulating biochemicals in the dorsal horn and injured nerve, suppressing inflammatory cells, improving neurological function, and accelerating nerve regeneration. The synergistic effects of LIPUS combined with methylcobalamin on neuroprotection were supported by the present findings.

Some limitations should be taken into account. First, the brief period of single treatment (LIPUS or methylcobalamin alone) used in the present study, while sufficient to promote sensory recovery and modest functional recovery, is not optimal for maintaining functional recovery after therapy ceases. Although it does not result in deterioration, at least as measured during sensory behavioral tests, the enhancements of sensory function undoubtedly contribute to a restored ability of the animals to respond to different demands of stimuli sensation. Different paradigms including progressive dosage of treatment protocols having a more pronounced effect on motor recovery of distal limb merit further study. Second, this biochemical study assessed the underlying injured-induced neural modulation of LIPUS, methylcobalamin, and combined therapies in early-phase BPI within the peripheral and spinal cord levels. According to previous studies, chronic BPI may also involve neuropathic pain [47,48]. More advanced studies will be required to establish a chronic BPI model with sensory allodynia and neuropathic pain that can provide more information on such neuroplasticity alterations of peripheral and spinal biochemical modulation arising from single or combined uses of LIPUS and methylcobalamin in the management of BPI-induced neuropathic pain. Finally, this study lacks serial morphological assessment. Aspects related to the initiation of nerve regeneration, degeneration, and progression in patients with BPI must be considered before any final judgment can be made on therapeutic efficacy, which warrants further investigation.

## 4. Materials and Methods

### 4.1. General Design

**Figure 5:**
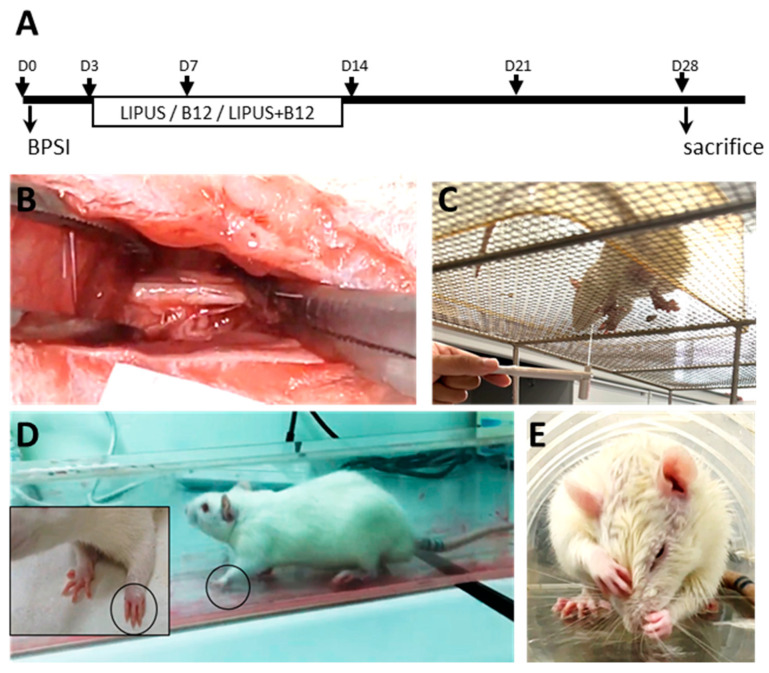
Overview of study design (**A**). Rats received brachial plexus stretching surgery ((**B**), Day 0, D0) and were then treated with low-intensity pulsed ultrasound (LIPUS) or methylcobalamin (B12) alone or in combination at 3 days after BPI surgery (Day 3, D3) for 12 days (ended at Day 14, D14). Assessments of functional recovery included sensory behavioral tests ((**C**), e.g., von Frey filament test) and motor function tests ((**D**), walking track analysis and (**E**), grooming test) and measured before surgery (D0), after surgery (D3) as well as at 7, 14, 21, and 28 days (D7, D14, D21, and D28) after surgery. Animals were sacrificed for immunoassays (immunohistochemistry and morphological studies) on Day 28.

Forty adult male rats were all subjected to BPI surgery at a randomly selected unilateral brachial plexus. They were then randomly divided into five groups: BPI animals (1) treated with LIPUS (*n* = 8, BL), (2) treated with methylcobalamin (*n* = 8, BB12), (3) treated with methylcobalamin combined with LIPUS (*n* = 8, BB12L), (4) without any treatments (*n* = 8, B group), and (5) those operated with sham-BPI without any treatments (*n* = 8, sB group). Three days after surgery, rats were assigned to LIPUS and/or methylcobalamin administration daily for 12 days. Assessments of functional recovery included the motor function tests and sensory behavioral tests measured before surgery (Day 0, D0), 3 days after surgery (before treatment, Day 3, D3), as well as 7, 14, 21, and 28 days (D7, D14, D21, and D28) after surgery. The rats were sacrificed after completion of the treatment for analysis of nerve morphology and immunohistochemistry. The experimental design and experimental groups are shown in Figure 5.

### 4.2. Animal Care and Preparation

Experiments were performed on adult male Sprague Dawley rats (SD, 250 to 300 g, purchased from BioLASCO Co., Ltd., Taipei, Taiwan). The experimental rats were housed and managed by the same animal breeder in standard animal rooms at 22 °C. All rats were allowed free access to food pellets and water. The feeding and management conditions were consistent for each group. Upper limb use and mobility were normal before surgery. Each animal was housed individually and cared for following the ethical guidelines of the International Association for the Study of Pain in Animals [49]. Effort was made to minimize discomfort and to reduce the number of animals used. All animal experiments were conducted following procedures approved by the Animal Care and Use Committee of China Medical University in accordance with the Guidelines for Animal Experimentation (CMUIACUC-2020-031).

### 4.3. Surgical Procedures for Brachial Plexus Stretching Injury

This study adopted the BPI model for rats, modified originally by Wall et al. [50]. Briefly, animals were placed inside the anesthesia chamber and oxygen was then turned on to flow at 1–2 L per minute. Animals were then anesthetized with 2% isoflurane (AErrane, Baxter Healthcare of Puerto Rico, Guayama, PR, USA) using a Veterinary Anesthesia Vaporizer (VIP 3000™, Matrx, Cincinnati, OH, USA). Upon losing the righting reflex, rats were removed from the chamber and placed onto a nosecone with a 0.5% maintenance dose of isoflurane. During anesthesia, parameters that should be monitored in an anesthetized rat include anesthetic depth by the response to toe pinch or the surgical stimulus, respiratory rate and pattern, heartbeats, mucous membrane color, and body temperature (between 96.5 and 99.5 °F) [51]. The brachial plexus was approached through a horizontal incision parallel to the clavicle, running from the sternum to the axillary region (1 cm approximately). In groups with BPI, the upper trunk was grasped with forceps at the location of 1 cm from the spinal cord and stretched by 12% of its length. The strain was maintained for one minute. Nerve conduction was monitored during the period of stretch and for a one-hour recovery period. In the sham-BPI group, the brachial plexus was exposed without any stretch or lesion to the nerve. The tissue layers were then brought together, and the skin was closed with a 4-0 silk suture string (Ethicon, Edinburgh, UK). Cefazolin (15 mg/kg, Taiwan Biotech Co., LTD., Taoyuan, Taiwan) and levobupivacaine (2.5 mg/kg, Abbott Laboratories Services Corp, Taiwan Branch, Taiwan) were administered into the surgical wound for controlling postoperative pain and infection for two days [52].

### 4.4. Intervention

#### 4.4.1. Low-Intensity Pulsed Ultrasound

LIPUS was applied with a pulsed mode of ultrasound at a frequency of 1.0 MHz, irradiation intensity of 0.1 W/cm^2^, and a 20% duty cycle for 20 min per treatment session for 12 days. These ratios resulted in burst length and burst period of 2 ms and 8 ms, respectively. Sonication was produced using a commercially therapeutic ultrasound physical therapy device (US-750, ITO Co., Tokyo, Japan) with a treatment head of 1.7 cm in diameter and an effective radiating area of 0.75 cm^2^. Transcutaneous LIPUS stimulation was applied in a fixed manner to the skin where the injured brachial plexus was located.

#### 4.4.2. Methylcobalamin Administration

Under ultrasonic guidance (16HL7, Terason t3000™ Ultrasound System, Ormond Beach, FL, USA), the syringe needle for injection of methylcobalamin (1 mg/kg; Methylcobal^®^ Eisai, Tokyo, Japan) was inserted into the BPI region at an angle of approximately 60 degrees. Daily local injection of methylcobalamin into the injured nerve area was administered for 12 days. For the combination of LIPUS and methylcobalamin treatment group (BB12L group), daily methylcobalamin injection was applied followed by LIPUS treatment for 12 days as described above.

### 4.5. Functional Assessments

All functional and behavioral tests were conducted at room temperature (27 °C) between the hours of 09:00 and 16:00 by one experimenter who was blinded to the treatment condition. Rats were placed in acrylic cages (32 cm × 22 cm × 27 cm) with a wire grid floor in a quiet room for 30 min of habituation prior to testing.

#### 4.5.1. Sensory Behavioral Assessments

(1) Cold sensitivity

Cold sensitivity was assessed using the acetone stimulation test. Fifty microliters of acetone (Honeywell Burdick & Jackson, Muskegon, MI, USA) were sprayed onto the plantar skin of each hind paw. The onset threshold (latency in seconds) of withdrawal responses evoked by acetone stimulation was determined as the cold withdrawal threshold (s), including foot lifting, shaking, licking, and squeaking. Paw movements associated with weight shifting or locomotion were not counted. The cut-off time was set at 20 s, at which point the animal was removed from the apparatus regardless of behavioral response. Acetone stimulation was repeated 3 times at intervals of 5 min for each test, and the mean onset paw withdrawal threshold was calculated.

(2) Thermal sensitivity

To examine thermal hyperalgesia according to the method used by Hargreaves et al. [53], the rat was placed on the surface of a 2 mm thick glass plate covered with the same plexiglass chamber to measure the sensitivity to heat stimuli. The lateral plantar surface of the injured forepaw was exposed to a constant-intensity radiant heat source (focused beam of light, beam diameter: 0.5 cm, intensity: 20 I.R.) through a transparent Perspex surface and a Plantar Analgesia Meter (IITC Model 400 Plantar Analgesia Meter, IITC Life Science Inc., Los Angeles, CA, USA). The onset threshold (latency in seconds) of withdrawal responses from the start of the radiant heat stimulation was recorded as the thermal paw withdrawal threshold (s). The cut-off time set at 20 s was used to prevent tissue damage. Three trials for each test were performed at 5 min intervals and the mean onset paw withdrawal threshold was calculated.

(3) Mechanical sensitivity

Mechanical sensitivity was assessed by measuring the mechanical paw withdrawal thresholds to pressure stimulation with an electronic von Frey aesthesiometer (Part # 2390, IITC Life Science Inc., Los Angeles, CA, USA) as described previously [27,54]. A non-anesthetized rat was placed in a small acrylic box of a confined area on wire mesh to limit its action. The front paw of the rat was poked with a special Rigid Tip (Rigid Tip + 0.01-inch tungsten electrode) to measure the mechanical threshold that induced withdrawal reflex response. The weakest pressure that elicited a response was taken as the mechanical paw withdrawal threshold (g). The final value for the response was obtained by averaging measurements from three trials.

#### 4.5.2. Motor Function Assessments

All experimental rats were submitted to the grooming test and walking track analysis for evaluating the motor function in the proximal and distal parts of the injured front limb. The grooming test that involved projecting a bowl of water over the animal’s head was performed in the cages. For walking track analysis, their forepaws were immersed in methylene blue, and they then had to walk through a tunnel of 15 cm × 120 cm in order to obtain regular tracks. Three measurements were taken and then averaged to determine the animal’s motor function of the front limbs. The performance in the grooming test for evaluation of the proximal part of the front limb’s motor function was scored as follows. (1) The animal’s forepaws only reach the mouth or elbow extension contracture. (2) The paws can touch the mouth and the region just under the eyes. (3) The paws can reach the eyes. (4) The paws can reach the front of the ears but not the back. (5) They can reach the back of the ears [55]. The finger spread (FS) and the maximal print length (PL) were determined using the forepaw prints. FS was the distance measured between the first and fourth digits. The first digit was considered the one closest to the thenar pad. The exact point for measurement was the outside of the fingertip print, not including the nail marks, smudged prints from sliding the paw on the paper, or ink spreads. PL was the distance measured between the third digital tip print and the outside limit of the thenar pad print at the wrist [55]. The FS/PL index was indicative of forepaw motor function.

### 4.6. Morphology and Immunoassays

#### 4.6.1. Tissue Preparation and Morphological Examination

All animals from each group were euthanized by anesthetic overdose one day after the final treatment session. The brachial plexus and the C5-T1 of spinal cords were harvested and fixed in 4% paraformaldehyde for 2 h at 4 °C and then embedded in paraffin. For the light microscopy analysis, spinal cord and nerve sections of 5 μm thickness in transverse and sagittal planes with a microtome were stained with hematoxylin-eosin by a light microscope for analysis with hematoxylin and eosin (H&E) staining to identify the morphology of injured nerves. The area of the inflamed cell and nerve nuclei (%) and vacuole (%) in the H&E-stained sections were measured.

#### 4.6.2. Immunohistochemical Staining and Quantitative Analyses

Immunohistochemical staining assay was examined in five alternate sections of spinal cord and nerve specimens, which were selected at a periodic sampling interval of four sections for analysis. The slides of brachial plexus sections were first incubated overnight at 4 °C with polyclonal rabbit anti-S100 primary antibody (1:400; S2644, Sigma-Aldrich, St. Louis, MO, USA) and antibody against BDNF (1:500, ab108319, abcam, Grove St, MA, USA). The spinal cord sections were incubated at 4 °C with antibody against SP (1:2000, #20064, ImmunoStar, Hudson, WI, USA) and iba1 (1:200, # PA5-27436, Thermo Fisher Scientific, Waltham, MA, USA), followed by incubation with biotinylated goat anti-rabbit IgG secondary antibody (Jackson ImmunoResearch Laboratories, Inc., West Grove, PA, USA) and a streptavidin-horseradish peroxidase conjugate (Jackson ImmunoResearch Laboratories, Inc., West Grove, PA, USA). Finally, the sections were visualized as brown precipitates by adding 3, 3′-diaminobenzidine (DAB, Pierce, Rockford, IL, USA) as a substrate. Negative control sections received the same treatment without the addition of primary antibodies. Sections were examined under a light microscope (BX43, Olympus America Inc., New Hyde Park, NY, USA) and photographed using a digital color camera (Auto exposure mode, DP70, Olympus America Inc.) in five randomly selected fields of the dorsal horn regions of C5-T1 spinal cords and nerve picked by an investigator (YNP or YCC) who was blinded to the experimental condition. Digital images were analyzed by computer-based morphometry using the ImageScope software 12.4.6 package with the Color Deconvolution v9 tool (v9.1.19.1571, Aperio, Vista, CA, USA). The percentages of strong positive pixels (upper-intensity limits of RGB value at 170) to total area pixels of selected nerve or spinal cord quantified SP-, iba1-, BDNF-, and S100-like immunoreactivities (SP-LI, iba1-LI, BDNF-LI, and S100-LI).

### 4.7. Statistical Analysis

All data are expressed as mean ± standard deviation (SD). The Kolmogorov–Smirnoff test indicated a non-normal distribution of all data in all measures. The effects of treatment on functional assessments, quantitative analyses of morphological and immunohistochemical studies in contents of inflammatory cells, S100 and BDNF in injured nerves, as well as SP and iba1 in dorsal horns of spinal cords, were each examined with a Kruskal–Wallis test to determine the significant differences among the five groups (BL, BB12, BB12L, B, and sB). Post hoc comparisons between two groups were analyzed with the Mann–Whitney U test. A Friedman test was performed to determine the differences among the six time points (D0, D3, D7, D14, D21, and D28) in each group and a post hoc analysis was conducted using the Wilcoxon signed-rank test. A *p* value of <0.05 was considered statistically significant. All data were analyzed using SPSS version 22.0 for Windows (IBM Company, Armonk, NY, USA).

## 5. Conclusions

BPI carries serious ramifications in terms of permanent disability of a paralyzed extremity, prolonged recuperation, and significant socio-economic impact. Findings from this study confirmed that after BPI, a series of physiological and pathological changes occur in the spinal cord, injury site, and target organs, leading to dysfunction. This research found improvement in sensory and motor functions and reduction in neuroinflammation, spinal microglia overactivation, and motor and sensory disturbance after the early intervention of LIPUS, methylcobalamin, and their combination in the BPI animal model, indicating that BPI-induced dysfunction can be modulated by these treatments. Moreover, it is the first study demonstrating the synergistic effects of early treatment with LIPUS and methylcobalamin combined, which can offer beneficial synergistic management for the initiation of early recovery after BPI. Identification of the critical features of LIPUS combined with methylcobalamin that facilitate early nerve regeneration is of importance and necessity. Further biochemical studies may reveal a central nociceptive transmission mechanism that can provide more information on such neuroplasticity alterations arising from the effects of combined therapy on dysfunction and impairment induced by BPI.

## Figures and Tables

**Table 1:** Results of serial alterations on functional assessments in the rat’s injured front limb of five groups and at six time points.

		Group					^#^ * Difference among Groups*	
		B (*n* = 8)	BL (*n* = 8)	BB12 (*n* = 8)	BB12L (*n* = 8)	sB (*n* = 8)	χ^2^ (4)	*p* Value
*Sensory withdrawal threshold* *								
1. Cold stimuli (sec)	D0	0.79 ^a^ (0.54–0.93)	0.83 ^a^ (0.44–0.99)	0.66 ^a^ (0.56–0.69)	0.64 ^a^ (0.51–0.69)	0.65 ^a, *NS*^ (0.53–0.91)	6.51	0.164
	D3	20.00 ^a^ (20.00–20.00)	20.00 ^a^ (20.00–20.00)	20.00 ^a^ (20.00–20.00)	20.00 ^a^ (20.00–20.00)	0.59 ^b, *NS*^ (0.52–0.92)	38.37	<0.001
	D7	18.55 ^a^ (9.20–20.00)	16.19 ^a^ (9.02–20.00)	13.00 ^a^ (8.85–19.1)	7.87 ^b^ (6.28–8.81)	0.56 ^c, *NS*^ (0.45–0.65)	30.68	<0.001
	D14	10.34 ^a^ (0.44–18.85)	7.50 ^a^ (1.85–12.09)	5.50 ^a^ (1.29–14.74)	4.40 ^b^ (3.36–5.88)	0.62 ^c, *NS*^ (0.50–0.65)	18.17	0.001
	D21	5.48 ^a^ (4.42–6.55)	0.60 ^b^ (0.46–5.75)	0.56 ^b, *NS*^ (0.46–1.02)	0.49 ^b, *NS*^ (0.45–0.76)	0.68 ^b, *NS*^ (0.64–0.87)	20.71	<0.001
	D28	4.38 **^a^** (3.12–5.92)	1.26 ^b^ (0.66–1.78)	1.06 ^b^ (0.6–1.68)	0.66 ^c, *NS*^ (0.5–0.77)	0.64 ^c, *NS*^ (0.60–0.68)	32.45	<0.001
^†^* Differences among time points*; χ^2^ (5)		33.33	36.12	37.64	38.00	11.64		
*p value*		<0.001	<0.001	<0.001	<0.001	0.054		
2. Thermal stimuli (sec)	D0	10.01 **^a^** (8.09–11.67)	10.05 ^a^ (8.06–14.17)	9.56 ^a^ (8.39–10.85)	9.75 ^a^ (8.13–11.42)	10.25 ^a, *NS*^ (8.02–10.99)	7.07	0.132
	D3	20.00 ^a^ (16.96–20.00)	20.00 ^a^ (13.59–20.00)	19.81 ^a^ (18.00–20.00)	19.88 ^a^ (18.12–20.00)	10.55 ^b, *NS*^ (8.44–14.05)	20.47	<0.001
	D7	15.45 ^a^ (12.43–17.32)	13.98 ^b^ (11.51–20.00)	16.50 ^a^ (9.81–20.00)	11.79 ^b^ (6.99–17.17)	9.12 ^c, *NS*^ (6.93–10.90)	19.56	0.001
	D14	17.07 ^a^ (10.86–19.77)	11.10 ^b, *NS*^ (6.56–17.31)	10.61 ^a, *NS*^ (8.62–18.70)	9.98 ^b, *NS*^ (6.47–12.89)	9.09 ^c, *NS*^ (8.32–13.22)	20.58	<0.001
	D21	6.82 ^a^ (5.10–9.26)	9.32 ^b^ (6.64–10.35)	9.94 ^b, *NS*^ (8.35–11.76)	9.84 ^b, *NS*^ (8.23–11.51)	9.20 ^b, *NS*^ (7.91–11.93)	14.75	0.005
	D28	6.79 ^a^ (4.08–9.02)	8.29 ^b^ (7.09–9.75)	10.38 ^b, *NS*^ (9.38–11.24)	10.32 ^b, *NS*^ (9.06–11.00)	9.20 ^b, *NS*^ (7.43–10.36)	22.78	<0.001
^†^* Differences among time points*; χ^2^ (5)		36.57	27.72	28.44	31.14	8.21		
*p value*		<0.001	<0.001	<0.001	<0.001	0.145		
3. Mechanical stimuli (sec)	D0	10.44 ^a^ (9.14–11.84)	10.09 ^a^ (9.18–11.78)	10.76 ^a^ (8.00–11.22)	9.92 ^a^ (8.92–11.44)	9.67 ^a, *NS*^ (8.10–11.30)	2.20	0.699
	D3	20.00 ^a^ (20.00–20.00)	20.00 ^a^ (20.00–20.00)	20.00 ^a^ (20.00–20.00)	20.00 ^a^ (20.00–20.00)	9.59 ^b, *NS*^ (7.36–10.54)	38.37	<0.001
	D7	17.32 ^a^ (13.70–20.00)	15.95 ^b^ (7.34–18.00)	16.72 ^a^ (13.00–20.00)	13.74 ^b^ (8.90–15.08)	9.29 ^c, *NS*^ (8.90–10.26)	21.68	<0.001
	D14	16.81 ^a^ (7.60–18.58)	7.85 ^b, *NS*^ (4.34–18.30)	10.00 ^a, *NS*^ (6.60–16.80)	8.31 ^b, *NS*^ (6.64–14.96)	9.21 ^b, *NS*^ (7.90–10.58)	11.01	0.026
	D21	6.63 ^a^ (1.54–9.04)	8.37 ^b^ (5.96–9.24)	9.26 ^b, *NS*^ (4.26–9.82)	8.99 ^b, *NS*^ (7.82–11.06)	8.98 ^b, *NS*^ (7.36–10.12)	10.95	0.027
	D28	6.32 ^a^ (3.18–7.62)	8.79 ^b^ (5.86–9.62)	9.92 ^b, *NS*^ (8.50–10.92)	9.68 ^b, *NS*^ (8.20–11.64)	9.50 ^b, *NS*^ (8.16–10.64)	19.99	<0.001
^†^* Differences among time points*; χ^2^ (5)		36.34	26.93	33.96	26.64	2.28		
*p value*		<0.001	<0.001	<0.001	<0.001	0.810		
*Motor function examination*								
1. Grooming (score)	D0	5.00 ^a^ (5.00–5.00)	5.00 ^a^ (5.00–5.00)	5.00 ^a^ (5.00–5.00)	5.00 ^a^ (5.00–5.00)	5.00 ^a, *NS*^ (5.00–5.00)	NA	NA
	D3	1.00 ^a^ (1.00–1.00)	1.00 ^a^ (1.00–1.00)	1.00 ^a^ (1.00–1.00)	1.00 ^a^ (1.00–1.00)	5.00 ^b, *NS*^ (5.00–5.00)	39.00	<0.001
	D7	1.00 ^a^ (1.00–1.00)	1.00 ^a^ (1.00–2.00)	2.00 ^b^ (2.00–2.00)	2.00 ^b^ (1.00–3.00)	5.00 ^c, *NS*^ (5.00–5.00)	32.93	<0.001
	D14	3.00 ^a^ (2.00–3.00)	3.00 ^a^ (2.00–5.00)	3.00 ^b^ (3.00–4.00)	4.00 ^b^ (3.00–5.00)	5.00 ^c, *NS*^ (5.00–5.00)	25.23	<0.001
	D21	3.50 ^a^ (3.00–5.00)	5.00 ^b, *NS*^ (4.00–5.00)	5.00 ^b, *NS*^ (5.00–5.00)	5.00 ^b, *NS*^ (5.00–5.00)	5.00 ^b, *NS*^ (5.00–5.00)	21.73	<0.001
	D28	3.50 ^a^ (3.00–5.00)	5.00 ^b, *NS*^ (5.00–5.00)	5.00 ^b, *NS*^ (5.00–5.00)	5.00 ^b, *NS*^ (5.00–5.00)	5.00 ^b, *NS*^ 5.00–5.00)	22.22	<0.001
^†^* Differences among time points*; χ^2^ (5)		37.77	38.24	40.00	38.21	NA		
*p value*		<0.001	<0.001	<0.001	<0.001	NA		
2. Finger spreading (index)	D0	0.96 ^a^ (0.88–1.00)	0.96 ^a^ (0.89–1.00)	0.98 ^a^ (0.87–1.08)	0.95 ^a^ (0.90–1.07)	1.00 ^a, *NS*^ (0.89–1.02)	7.22	0.125
	D3	0.00 ^a^ (0.00–0.00)	0.00 ^a^ (0.00–0.00)	0.00 ^a^ (0.00–0.00)	0.00 ^a^ (0.00–0.00)	1.00 ^b, *NS*^ (0.92–1.08)	38.38	<0.001
	D7	0.00 ^a^ (0.00–0.00)	0.00 ^a^ (0.00–0.00)	0.00 ^a^ (0.00–0.00)	0.00 ^a^ (0.00–0.00)	0.98 ^b, *NS*^ (0.95–1.13)	38.39	<0.001
	D14	0.00 ^a^ (0.00–0.00)	0.00 ^a^ (0.00–0.000)	0.00 ^b^ (0.00–0.87)	0.00 ^b^ (0.00–0.71)	1.00 ^c, *NS*^ (0.93–1.02)	29.30	<0.001
	D21	0.74 ^a^ (0.52–0.79)	0.78 ^a^ (0.00–0.92)	0.78 ^b^ (0.73–0.98)	0.83 ^b^ (0.76–0.90)	0.98 ^c, *NS*^ (0.92–1.02)	23.78	<0.001
	D28	0.76 ^a^ (0.71–0.87)	0.77 ^a^ (0.66–1.03)	0.86 ^b^ (0.78–0.96)	0.91 ^b, *NS*^ (0.87–0.98)	0.98 ^c, *NS*^ (0.93–1.05)	25.23	<0.001
^†^* Differences among time points* χ^2^ (5)		38.79	36.69	37.57	38.35	3.90		
*p value*		<0.001	<0.001	<0.001	<0.001	0.564		

Data are presented as median, and range in parentheses; NA: Not Applicable. * measured by onset latencies of withdrawal behaviors; ^#^ tested by Kruskal–Wallis test; ^†^ tested by Friedman test; ^*NS*^: values with *p* > 0.05 indicate no significant difference between various time points and D0 (before brachial plexus stretching injury) tested by Wilcoxon sign rank test; a, b, c: values with different superscripts (e.g., a vs. b and b vs. c) indicate significant differences (*p* < 0.05) between two groups tested by Mann–Whitney test.

## Data Availability

The datasets generated during and/or analysed during the current study are available from the corresponding author on reasonable request.
